# FGF21 alleviates acute liver injury by inducing the SIRT1‐autophagy signalling pathway

**DOI:** 10.1111/jcmm.17144

**Published:** 2022-01-04

**Authors:** Xiaoning Yang, Zhongqian Jin, Danfeng Lin, Tianzhu Shen, Jiangnan Zhang, Dan Li, Xuye Wang, Chi Zhang, Zhuofeng Lin, Xiaokun Li, Fanghua Gong

**Affiliations:** ^1^ The Third Affiliated Hospital of Wenzhou Medical University Wenzhou China; ^2^ School of Pharmacy Wenzhou Medical University Wenzhou China; ^3^ The Second Affiliated Hospital of Wenzhou Medical University Wenzhou China

**Keywords:** autophagy, Beclin1, CCl_4_, FGF21, LC3 II, liver injury, SIRT1

## Abstract

Liver injury can lead to different hepatic diseases, which are the mainly causes of high global mortality and morbidity. Autophagy and Sirtuin type 1 (SIRT1) have been shown protective effects in response to liver injury. Previous studies have showed that Fibroblast growth factor 21 (FGF21) could alleviate acute liver injury (ALI), but the mechanism remains unclear. Here, we verified the relationship among FGF21, autophagy and SIRT1 in carbon tetrachloride (CCl_4_)‐induced ALI. We established CCl_4_‐induced ALI models in C57BL/6 mice and the L02 cell line. The results showed that FGF21 was robustly induced in response to stress during the development of ALI. After exogenous FGF21 treatment in ALI models, liver damage in ALI mice was significantly reduced, as well as serum alanine aminotransferase (ALT) and aspartate aminotransferase (AST) levels. Consistently, FGF21 also greatly reduced the levels of ALT, AST, pro‐inflammatory cytokines interleukin 6 (IL6) and tumour necrosis factor‐alpha (TNFα) in ALI cell lines. Mechanistically, exogenous FGF21 treatment efficiently upregulated the expression of autophagy marker microtubule‐associated protein light chain‐3 beta (LC3 II) and autophagy key molecule coiled‐coil myosin‐like BCL2‐interacting protein (Beclin1), which was accompanied by alleviating hepatotoxicity in CCl_4_‐treated wild‐type mice. Then, we examined how FGF21 induced autophagy expression and found that SIRT1 was also upregulated by FGF21 treatment. To further verify our results, we constructed an anti‐SIRT1 lentit‐RNAi to inhibit SIRT1 expression in mice and L02 cells, which reversed the protective effect of FGF21 on ALI. In summary, these results indicate that FGF21 alleviates ALI by enhancing SIRT1‐mediated autophagy.

## 1 INTRODUCTION

The liver is the most important metabolic organ in the human body; it is responsible for the metabolism of carbohydrates, proteins and lipids, and has important physiological functions in metabolism, oxidation and reduction.^1^ The liver is often subject to various types of damage, including viral infections, trauma and chemical agents, which usually lead to cell death and liver dysfunction. Chemical liver injury is the most common cause in clinical practice. It mainly includes environmental toxic liver injury, alcoholic liver injury and drug‐induced liver injury.^2^, ^3^ For drug‐induced liver injury, N‐acetylcysteine is the only effective treatment; however, there are often severe limitations such as adverse reactions and narrow therapeutic windows.^4^, ^5^ Therefore, it is crucial to deeply understand the pathogenesis of liver injury and screen for new drugs with hepatoprotective activity.

Fibroblast growth factor 21 (FGF21) is a secretory protein composed of 181 amino acids; it is expressed primarily by liver cells. Several studies have recently shown that FGF21 protects the liver when the body is subjected to acute stimuli that cause damages.^5^, ^6^, ^7^ Previous studies have demonstrated that levels of FGF21 in the liver and serum increased dramatically in an acetaminophen‐induced mouse liver injury model. Compared with wild‐type mice, FGF21‐knockout mice exhibited markedly elevated serum transaminase levels and increased liver necrosis, resulting in a significant increase in mortality. Meanwhile, the level of liver oxidative stress increased and the anti‐oxidant capacity decreased during the liver injury. In contrast, mice treated with recombinant FGF21 showed reversal of drug‐induced oxidative stress, as reflected by less neutrophil infiltration and liver injury.^8^, ^9^

Autophagy is a process of cellular self‐catabolism; its primary function is to remove unwanted cytoplasmic contents, including misfolded or aggregated proteins, accumulated lipids, excessive or damaged organelles, and harmful pathogens, through lysosome degradation to achieve the metabolic needs of the cell and the renewal of some organelles.^10^ Under physiological conditions, low levels of autophagy in hepatocytes are crucial for maintaining normal liver function.^11^ Under the influence of some stimuli, such as starvation or oxidative stress, the autophagic activity can be stimulated or inhibited. Animal experiments have demonstrated that autophagy‐deficient mice develop spontaneous hepatomegaly and liver injury.^12^ Experiments on acetaminophen overdose‐induced liver injury showed increased autophagy in hepatocytes, which led to the removal of damaged mitochondria. Moreover, rapamycin, an activator of autophagy, further enhanced the activity of autophagy, whereas the autophagy inhibitor chloroquine and autophagy‐related gene 7 (Atg7) deletion aggravated liver injury.^13^, ^14^ Previous studies have reported that fenofibrate protects against acetaminophen‐induced hepatotoxicity by regulating the expression of FGF21 and thereby regulating autophagy in mice.^15^ In our study, we found treated exogenous FGF21 protein could markedly upregulate expression of autophagy while FGF21 alleviates CCl_4_‐induced ALI.

Sirtuin type 1 (SIRT1), a histone deacetylase widely distributed in various metabolically important tissues (liver, skeletal muscle, pancreas and adipose tissue), profoundly affects homeostasis by regulating a variety of transcription factors.^16^ A previous study found that FGF21 improved alcoholic fatty liver disease by activating the AMPK‐SIRT1 signalling pathway.^17^ Regulation of FGF21/SIRT1 participates in the improvement of steatohepatitis and hepatic steatosis.^18^, ^19^, ^20^, ^21^ Another study found that fish oil alleviated liver injury induced by intestinal ischemia/reperfusion through the AMPK/SIRT1/autophagy pathway.^22^ Nevertheless, the molecular mechanisms of FGF21, autophagy and SIRT1 in ALI remain unclear.

Although it has been found that FGF21, autophagy and SIRT1 have been shown protective effects in response to liver injury, the relationship among FGF21, autophagy and SIRT1 in CCl_4_‐induced ALI is yet to be understood. Therefore, in this study, we focused on exploring the regulatory mechanism of FGF21 on SIRT1 and autophagy to alleviate liver injury. Our results indicate that FGF21 could alleviate CCl_4_‐induced acute liver injury through inducible expression of autophagy which is specifically mediated by SIRT1.

## 2 MATERIALS AND METHODS

### 2.1 Animals and reagents

Male C57BL/6 mice (6–8 weeks old, supplied by the Model Animal Research Center of Nanjing) were reared in an SPF barrier of the animal experimental centre of Wenzhou Medical University. Room temperature was 23 ± 1°C, and humidity was 60% under 12 h/12 h light/dark cycle conditions. The mice were fed adaptively for 1 week before the experiment. Animals were restricted the access to food 12 h before modelling, but they continued having access to water. All of the animals were fed and housed in accordance with the guidelines of the National Institutes of Health of China.

### 2.2 Grouping and administration of drugs

The experiment was divided into two parts: first, mice were randomly divided into the control group, the CCl_4_‐treated group (ALI), the low‐dose FGF21 treatment group (L‐FGF2), the medium‐dose FGF21 treatment group (M‐FGF2) and the high‐dose FGF21 treatment group (H‐FGF2), with seven mice in each group. The ALI group was established by intraperitoneal injection of olive oil containing 10% CCl_4_ (Zhanyun Chemical) at 5 ml/kg. Two hours after modelling, the control group and the ALI group were injected with the same amount of normal saline. The L‐F21, M‐F21 and H‐F21 groups were intraperitoneally injected with 0.25 mg/kg, 0.5 mg/kg and 1 mg/kg recombinant FGF21 (Wenzhou Medical University) respectively. The control group was given an intraperitoneal injection of the same amount of olive oil. The mice were sacrificed 24 h later. Second, mice were randomly divided into a control group, a CCl_4_‐treated group (ALI), an FGF21‐treated group (ALI + FGF21) and a SIRT1‐knockdown group (siSIRT1 + FGF21), with seven mice in each group. The treatment of the control, ALI and FGF21‐treated groups was the same as in the first part. The SIRT1‐knockdown group was intraperitoneally injected with LV‐SIRT1‐RNAi (Genechem) at 1.6 × 109 TU kg^−1^ with SIRT1‐interfering lentivirus (4 × 108 TU/mouse). One week later, this group was treated in the same way as the FGF21‐treated group. All of the mice were sacrificed 24 h after CCl_4_ treatment.

### 2.3 Morphological observation

Liver tissues were fixed in 10% formalin, embedded in paraffin, cut into 4‐μm sections, stained with haematoxylin and eosin and examined under an optical microscope.

The liver tissues were fixed in 2.5% glutaraldehyde phosphate buffer and then fixed in 1% osmium acid. After dehydration, the liver tissues were repaired, ultrathin sectioned and stained with 4% uranium acetate and lead citrate. Finally, the sections were observed and photographed under a TECNAI 10 transmission electron microscope.

### 2.4 Determination of ALT, AST, and FGF21

Blood samples were centrifuged at 4°C, 3000 r/min for 15 min, and the supernatants were stored at −80°C until use. Serum ALT and AST levels were measured with their assay kits (Nanjing Jiancheng Bio). Serum levels of FGF21 were measured using an FGF21 ELISA kit (Abcam).

### 2.5 Immunohistochemical method

We incubated 5‐µm paraffin sections of the liver with anti‐FGF21 antibody (Abcam), anti‐SIRT1 antibody (Abcam), anti‐LC3 II antibody (Abcam) and Beclin1 antibody (Abcam) followed by incubation with secondary antibody and DAB (ZSGB Bio). The distribution and content of FGF21, SIRT1, LC3 II and Beclin1 in the liver were analysed by scanning 10–20 fields randomly selected under an upright microscope.

### 2.6 Immunofluorescence of tissue

We subjected 5‐µm paraffin sections of mouse liver to high‐pressure antigen retrieval using citrate and then blocked them in bovine serum albumin. Next, the tissues were incubated with anti‐Beclin1 and anti‐LC3 II antibodies overnight at 4°C. Next, we incubated the sections in goat anti‐rabbit antibodies conjugated to fluorescent probes at room temperature for 1 h and stained with DAPI (Abcam). Finally, the images were captured for analysis using a laser confocal microscope (Nikon, Ti‐E, and A1 plus).

### 2.7 Cell culture and administration

Normal human hepatocytes (L02) were cultured in high‐glucose DMEM medium containing 15% foetal bovine serum (FBS, Gibco) at 37°C in humid air containing 5% CO_2_. After various doses of CCl_4_ (2.5, 5, 10, 12.5 and 15 mmol/L) and recombinant FGF21 (50, 100 and 200 ng/ml) had been given, the cells were lysed and centrifuged (12,000 g, 15 min) to collect supernatants, which were then stored at −80°C. L02 cells were transfected with small interfering RNA (siRNA) targeting SIRT1, and a negative control group was generated as well. After 72 h from siRNA transfection, the cells were given the appropriate concentrations of CCl_4_ and FGF21 and then harvested for protein analysis.

### 2.8 Western blot analysis

Liver tissue or intracellular protein was extracted using reagents for extracting total proteins from mammalian tissues or cells and quantified using a BCA protein detection kit. The proteins were separated using sodium dodecyl sulphate‐polyacrylamide electrophoresis and transferred to PVDF membranes (Bio‐Rad). The membranes were sealed with TBST buffer containing 10% skimmed milk powder for 1.5 h at room temperature, then incubated with anti‐FGF21 antibody (1:1000), anti‐SIRT1 antibody (1:1000), anti‐LC3 II antibody (1:2000), anti‐Beclin 1 antibody (1:1000), anti‐β‐actin antibody (1:1000) or anti‐GAPDH antibody (1:1000) in a shaker at 4°C overnight. After washing three times with TBST, the membranes were incubated with a secondary antibody conjugated with horseradish peroxidase for 1 h. Protein signals were detected using a protein gel imaging system (chemiDocXRS + Imaging System Bio‐Rad, Universal Hood II).

### 2.9 Statistical analysis

All of the data were expressed as mean ± standard error of the mean (SEM). One‐way ANOVA and *t* tests were used to compare the groups. All of the data were analysed using GraphPad Prism 5.0 software. Differences were statistically significant when *p* was lower than 0.05.

## 3 RESULTS

### 3.1 Expression of FGF21 increased sharply during CCl_4_‐induced acute liver injury

**FIGURE 1:**
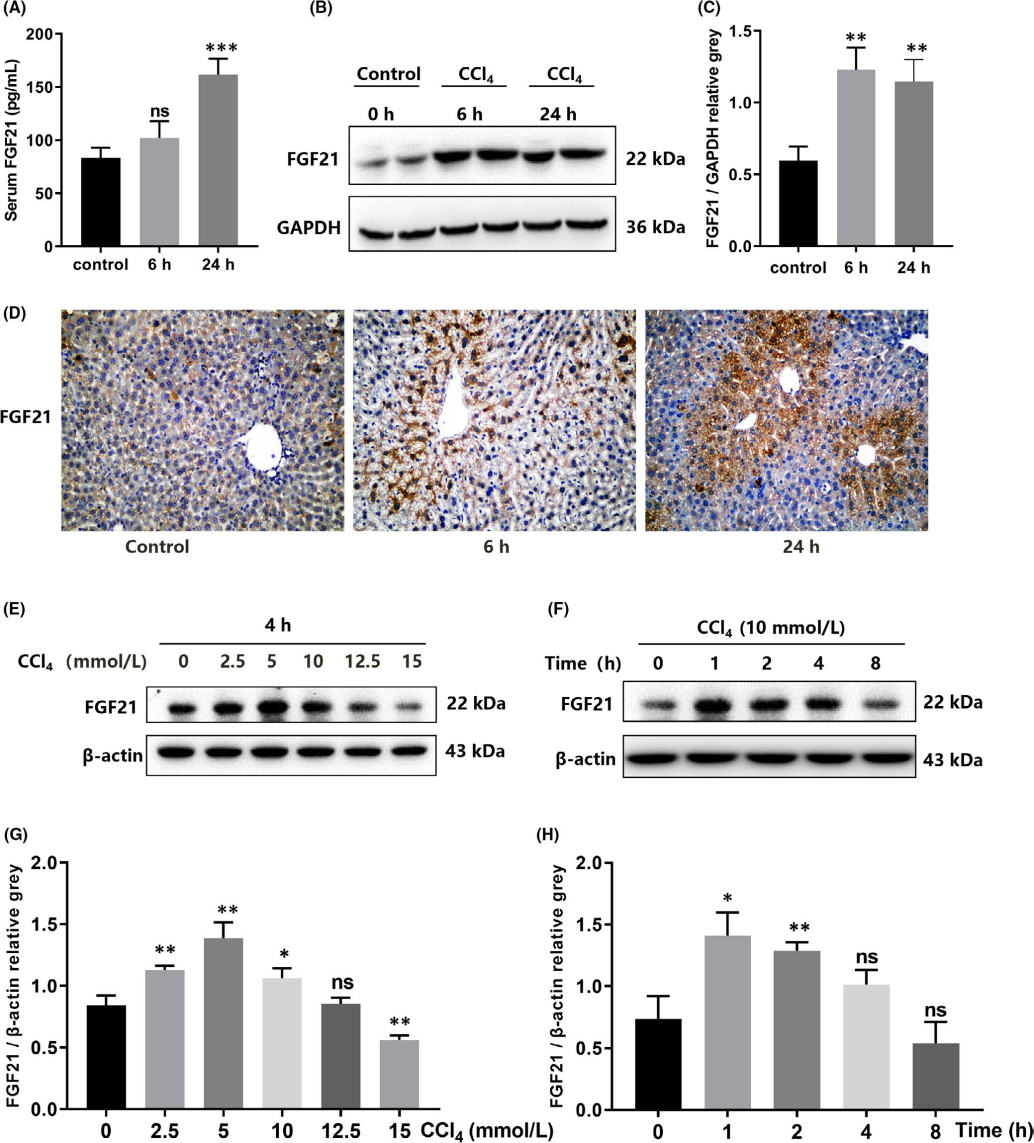
Upregulation of FGF21 expression during ALI. (A) Changes in FGF21 expression in serum at 6 h and 24 h after CCl_4_ injection. (B and C) The expression level of FGF21 in the liver by western blot. (D) Immunohistochemical results of FGF21 in the liver after 6 and 24 h of CCl_4_ treatment (scale bar = 50 µm). (E and G) Changes in FGF21 expression in normal hepatocytes L02 treated with different concentrations of CCl_4_ for 4 h. (F and H) Changes in FGF21 expression in normal hepatocytes L02 after administration of 10 mmol/L CCl_4_ at different time points. *p < 0.05, **p < 0.01, ***p < 0.001

Given the important role of FGF21 in protecting Liver, we first determined changes in FGF21 expression after ALI. After establishing an acute liver injury model in mice using CCl_4_, we found that serum transaminase levels increased sharply at 6 h and 24 h compared with the control group (Figure S1A, B). Haematoxylin and eosin staining and transmission electron microscopy showed that livers in the CCl_4_ group were severely damaged, as reflected by an irregular arrangement of liver cells, vacuolar degeneration or necrosis of large number of liver cells (Figure S1C). Liver organelles were also severely damaged, which was reflected by irregular nuclei, shrinkage of the nuclear membrane, chromatin pyknosis, organelle aggregation, uneven distribution of organelles, disappearance of mitochondrial ridge and enlargement of the endoplasmic reticulum (Figure S1D). In the ALI model, we measured FGF21 expression in serum and liver tissue. Serum levels of FGF21 were significantly higher than those of the control group, 1.20‐fold and 1.93‐fold at 6 h and 24 h respectively. The difference at 24 h was significant (Figure 1A). The levels of FGF21 in the ALI group were significantly higher than those in the control group, 2.06‐fold and 1.92‐fold at 6 h and 24 h respectively (Figure 1B,C). Immunohistochemical assays showed that the expression of FGF21 was concentrated in the most severely injured tissue (Figure 1D). Next, we performed *in vitro* assays to measure the levels of FGF21 in normal hepatocytes and L02 cells after injury. L02 cells were treated with increasing concentrations of CCl_4_ (2.5, 5, 10, 12.5 and 15 mmol/L). FGF21 expression in the ALI group increased gradually with the increasing doses of CCl_4_, peaking at 5 mmol/L and then decreasing (Figure 1E,G). We used 10 mmol/L CCl_4_ given at various time points (0, 1, 2, 4, 8 and 12 h). The levels of FGF21 increased most markedly at 1 h and 2 h after stimulation, then gradually decreased (Figure 1F,H). These findings suggest that FGF21 levels may increase in a compensatory manner for a short time in the context of ALI.

### 3.2 FGF21 alleviates CCl_4_‐induced acute liver injury

**FIGURE 2:**
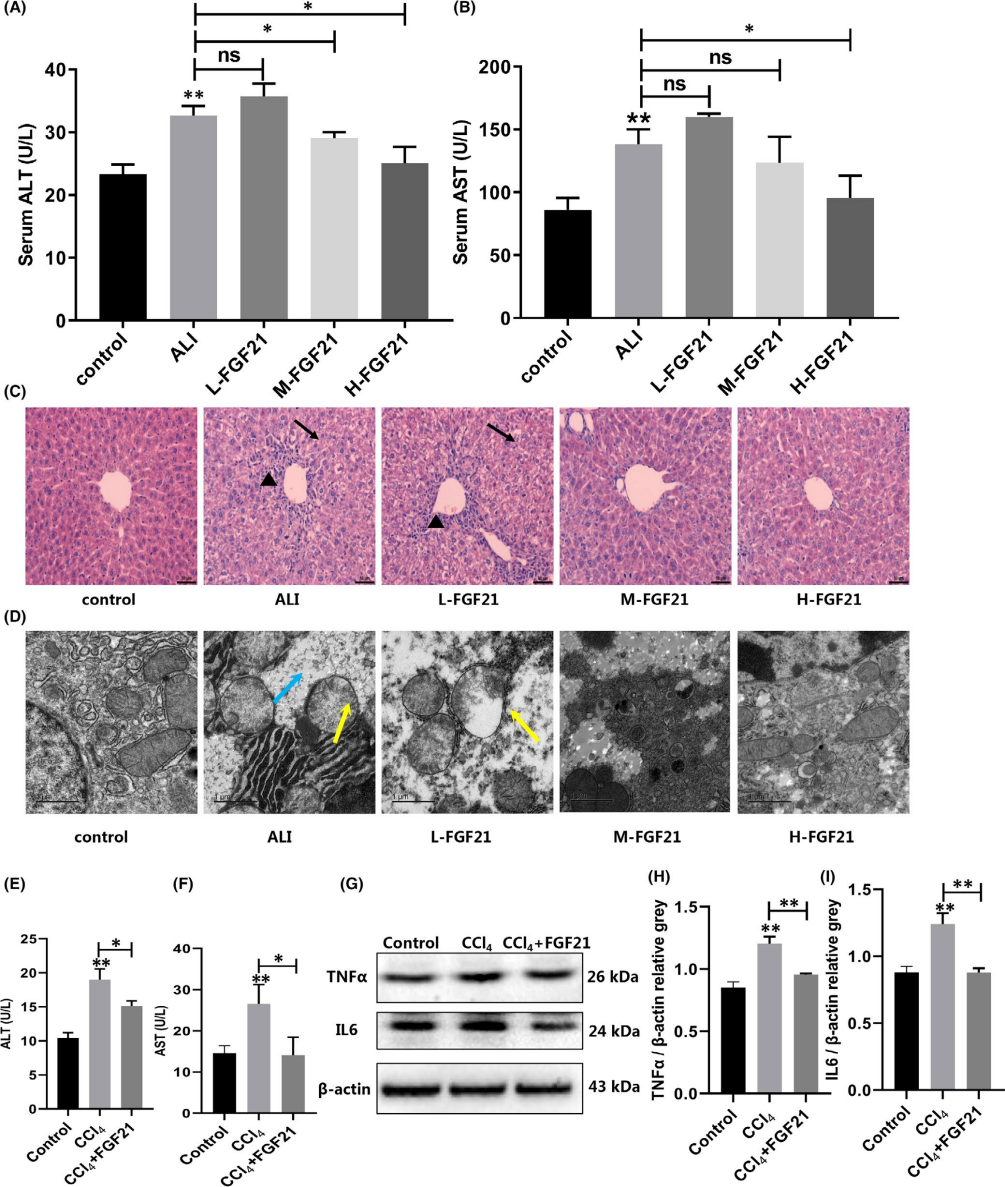
Exogenous recombinant FGF21 alleviated ALI. (A and B) Serum transaminase expression in mice injected with CCl_4_ for 2 h and after that with various doses of exogenous recombinant FGF21. (C) Haematoxylin and eosin staining of the liver after administration of various doses of recombinant exogenous FGF21 (scale bar = 50 µm). (D) The organelles' changes in hepatocytes were detected by transmission electron microscopy after administration of various doses of exogenous recombinant FGF21 (scale bar = 1 µm). (E and F) Transaminase expression in the L02 cells treated with CCl_4_ after administering 100 ng/mL exogenous recombinant FGF21 for 2 h. (G–I) The expression of IL6 and TNFα in the L02 cells treated with CCl_4_ after administering 100 ng/ml exogenous recombinant FGF21 for 2 h. *p < 0.05, **p < 0.01, ***p < 0.001

We next administered exogenous FGF21 to ALI mice and L02 cells. Assays of serum biochemical indexes (Figure 2A,B) showed that transaminase levels in the FGF21‐treated group were significantly lower. It is worth noting that the H‐FGF21 group decreased to levels close to those of the normal group. Haematoxylin and eosin staining showed that the degree of liver injury in L‐FGF21, M‐FGF21 and H‐FGF21 groups was lower than that in the ALI group. The liver structure recovery was most apparent in the H‐FGF21 group, with minimal infiltration of inflammatory cells. Simultaneously, a small amount of oedema and partial necrosis were seen in liver tissue of the low‐dose group (Figure 2C). Transmission electron microscopy revealed larger fat droplets in hepatocytes of the L‐FGF21 group; the organelles were evenly distributed, and most of the mitochondrial ridges were blurred or invisible (Figure 2D). In hepatocytes of the M‐FGF21 and H‐FGF21 groups, some mitochondria were pyknotic. Compared with the ALI group, the mitochondrial consolidation of hepatocytes was reduced after FGF21 administration, and the lipid droplets were significantly reduced, especially the H‐FGF21 group showed the best improvement.

We also examined the effect of FGF21 in vitro. L02 cells were stimulated with various concentrations of CCl_4_ (2.5, 5, 10, 12.5 and 15 mmol/L) for 4 h, while 5 mmol/L CCl_4_ was selected to stimulate the L02 cells at several time points (0, 1, 2, 4, 8 and 12 h). By detecting the expression levels of transaminases and inflammatory cytokines, tumour necrosis factor‐alpha (TNF α) and interleukin‐6 (IL‐6) in the cells of the ALI group (Figures S1E–J), the following optimal modelling conditions for causing acute liver injury to cells in vitro was determined: treatment with 10 mmol/L CCl_4_ for 4 h. The ALI group was given 100 ng/ml of FGF21 for 2 h. Transaminase levels in the ALI group were significantly lower by 1.25–1.88 times (Figure 2E,F). TNFα and IL6 levels in the ALI group were also significantly lower by 1.25 and 1.41 times respectively (Figure 2G–I). Based on the in vivo and in vitro assays, we believe that FGF21 improves CCl_4_‐induced ALI.

### 3.3 Autophagy is upregulated during FGF21 alleviates CCl_4_‐induced acute liver injury

**FIGURE 3:**
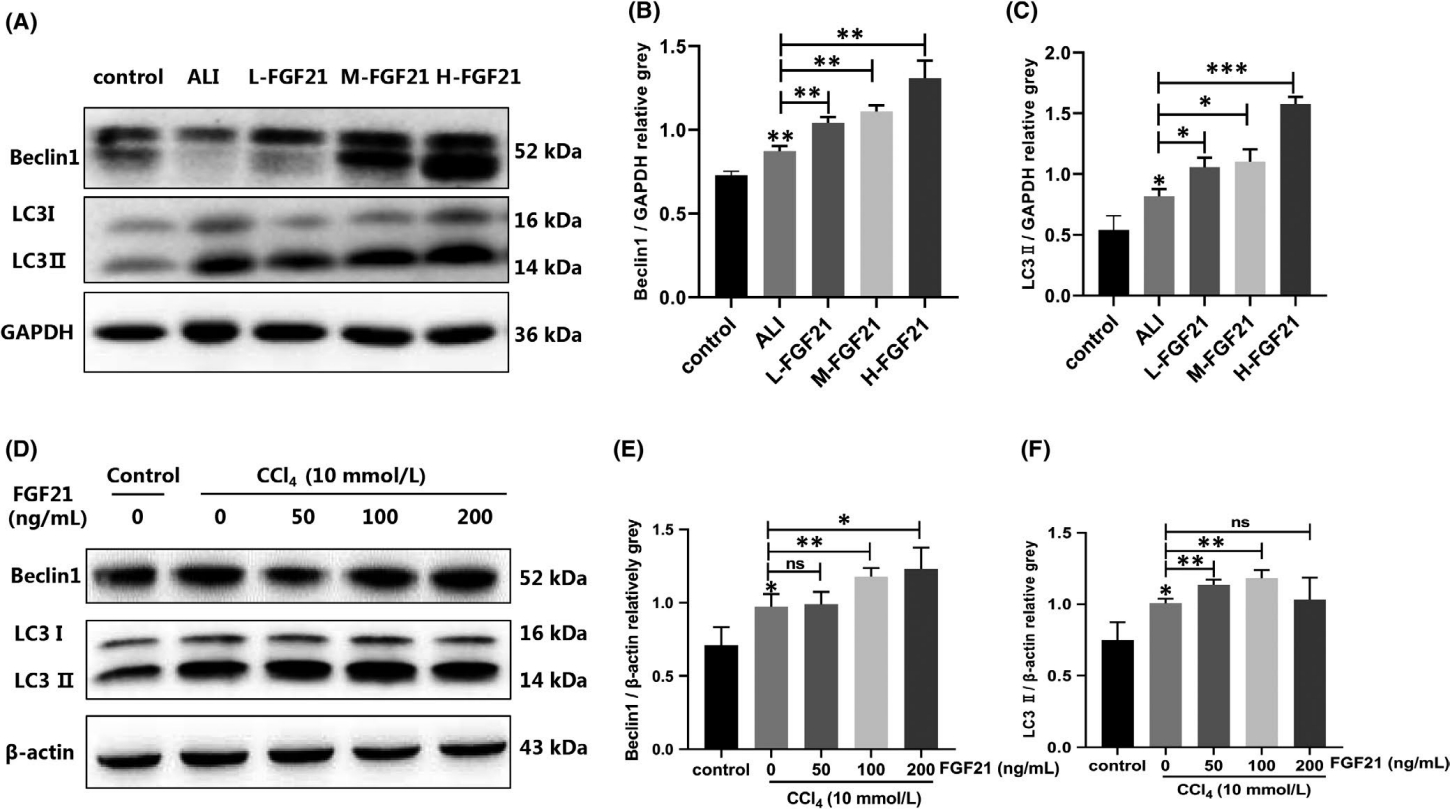
Autophagy was induced during FGF21 alleviation of ALI. (A–C) Mice were injected with CCl_4_ for 2 h, followed by various doses of recombinant exogenous FGF21. Then, we measured the expression of Beclin1 and LC3 II in the liver. (D–F) L02 cells treated with CCl_4_ were exposed to various doses of exogenous recombinant FGF21, and the expression levels of Beclin1 and LC3 II were measured (The beta‐actin band shown in (D) was the same as the one shown in Figure 4D because Beclin1 and LC3 II in (D) and SIRT1 in Figure 4D were run on the same blot.). *p < 0.05, **p < 0.01, ***p < 0.001

We next investigated the mechanism by which FGF21 improves ALI. Numerous studies have shown that autophagy protects against liver injury; therefore, we measured autophagy expression in the remission process mediated by FGF21. Compared with the control group, the expression levels of autophagy‐related proteins LC3 II and Beclin1 in livers of the ALI group were significantly greater. After giving various doses of FGF21, the levels of autophagy further increased, with the H‐FGF21 group showing the strongest effect. The levels of LC3 II and Beclin1 were significantly higher by 1.93 and 1.50 times respectively (Figure 3A–C). Similarly, in vitro assays showed that the expression levels of LC3 II and Beclin1 were further elevated in the ALI group after treatment with various concentrations of FGF21 (50, 100 and 200 ng/ml) for 2 h. The 100 ng/ml group had the most significant effect (Figure 3D–F). These findings suggest that the role of FGF21 in alleviating ALI is related to autophagy.

### 3.4 Inducible expression of SIRT1 during FGF21 alleviation of CCl_4_‐induced acute liver injury

**FIGURE 4:**
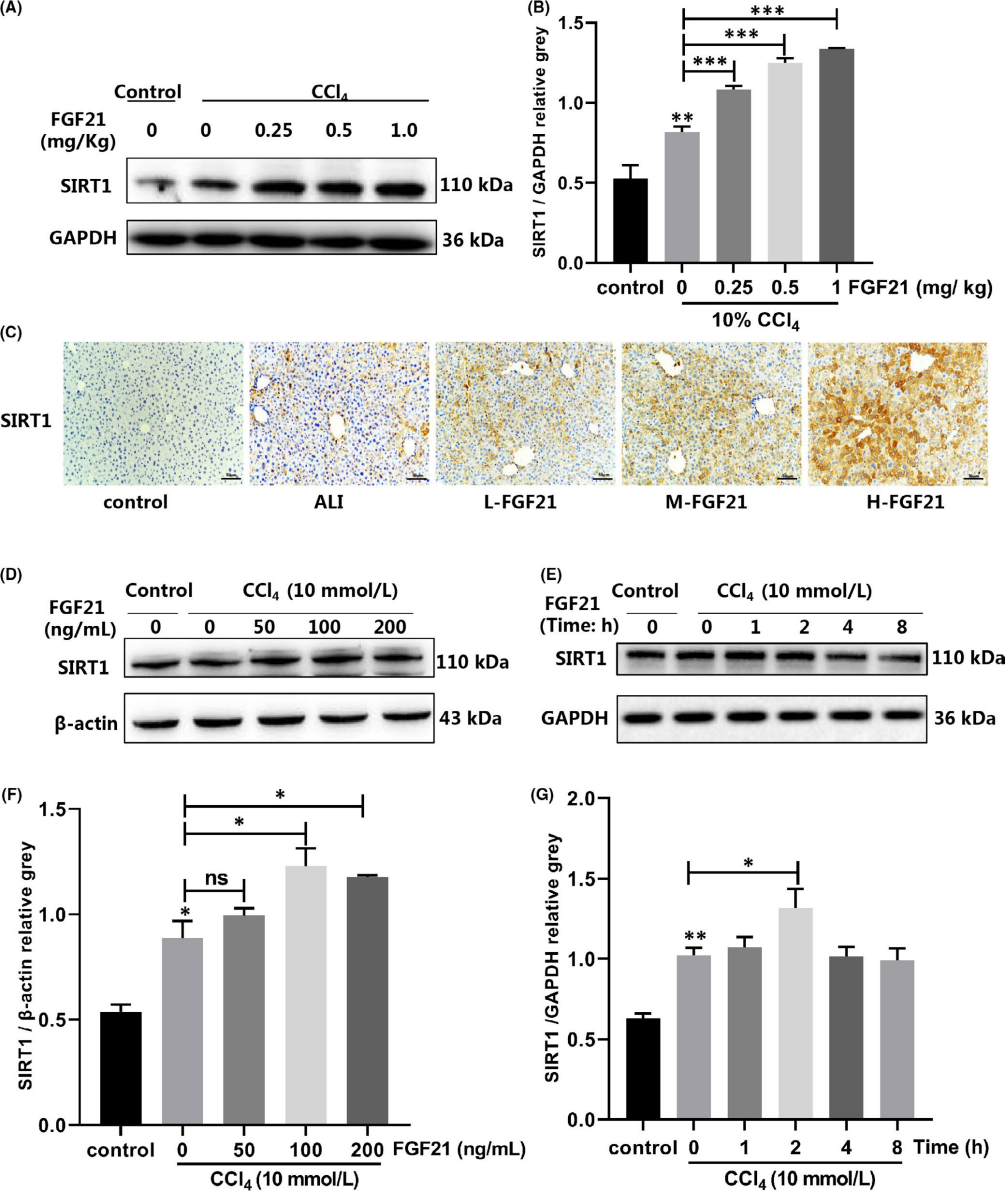
SIRT1 was upregulated in the process of alleviating ALI. (A and B) Various doses of recombinant exogenous FGF21 protein were given to the CCl_4_ injury group, and the expression of FGF21 in the liver was analysed using western blot (The beta‐actin band shown in (D) was the same as the one shown in Figure 3D because SIRT1 in (D) and Beclin1 and LC3 II in Figure 3D were run on the same blot.). (C) Various doses of recombinant exogenous FGF21 were given to the CCl_4_ injury group, and the immunohistochemical staining of SIRT1 in the liver was performed (scale bar = 50 µm). (D and F) SIRT1 expression in L02 cells treated with various concentrations of FGF21 for 2 h after CCl_4_ treatment. (E and G) L02 cells damaged by CCl_4_ were treated with 100 ng/ml FGF21 for various periods, and SIRT1 expression was determined. *p < 0.05, **p < 0.01, ***p < 0.001

Next, we explored how FGF21 regulates autophagy and alleviates ALI. Previous studies have shown that SIRT1 is involved in the activation of autophagy. We measured the expression levels of SIRT1 during the process of FGF21 alleviation of ALI. The expression of SIRT1 in the livers of the ALI group was significantly higher than that in the control group. SIRT1 levels increased after treatment with FGF21 in a dose‐dependent manner compared with the ALI group by 1.32, 1.52 and 1.63 times in L‐FGF21, M‐FGF21 and H‐FGF21 groups respectively (Figure 4A,B). Immunohistochemical staining of SIRT1 also showed that SIRT1 expression was higher, particularly in severely damaged areas (Figure 4C). We further confirmed the induction of SIRT1 by FGF21 in vitro. We gave various concentrations (50, 100 and 200 ng/ml) of FGF21 for 2 h and 100 ng/ml of FGF21 for various durations (1, 2, 4 and 8 h). SIRT1 levels were significantly upregulated by 1.38 times after treatment with 100 ng/ml FGF21 compared with the ALI group (Figure 4D,F). SIRT1 upregulation peaked (1.30‐fold) at 100 ng/ml of FGF21 after 2 h of treatment (Figure 4E,G).

### 3.5 FGF21 alleviates CCl_4_‐induced acute liver injury through SIRT1‐autophagy signalling pathway

**FIGURE 5:**
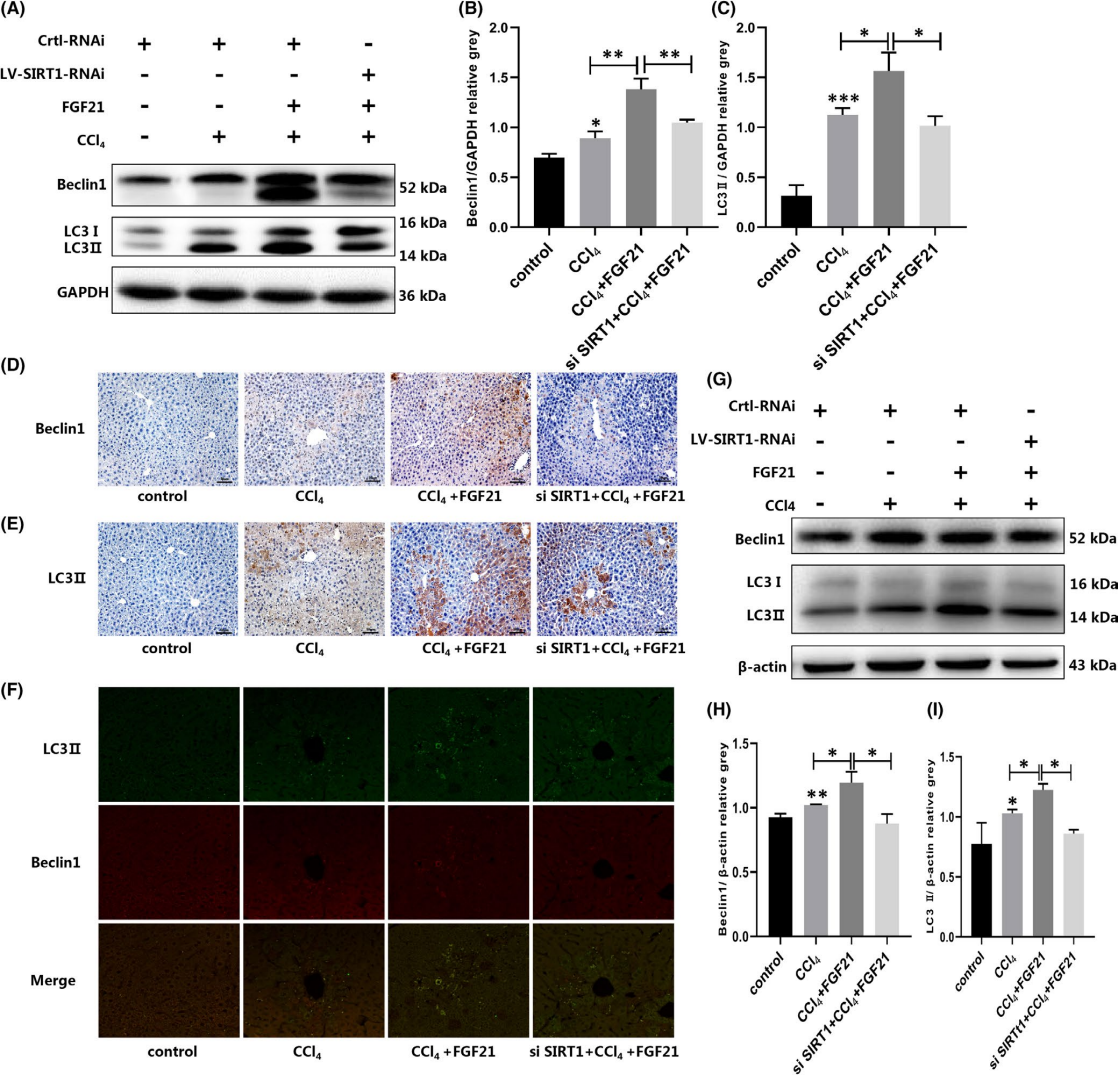
siRNA‐mediated SIRT1 downregulation in liver or hepatocytes significantly suppressed the FGF21‐related upregulation of autophagy. (A–C) Mice were injected with siRNA‐mediated SIRT1 lentivirus, then CCl_4_ and/or FGF21 were given to the corresponding groups. Beclin1 and LC3 II in the liver were assayed using western blot. (D and E) The immunohistochemical results of Beclin1 and LC3 II in the liver (scale bar = 50 µm). (F) Immunofluorescence staining of Beclin1 and LC3 II in the liver (scale bar = 50 µm). (G–I) L02 cells were transfected with the siRNA‐mediated SIRT1 lentivirus, then CCl_4_ and/or FGF21 were given to the corresponding groups. Next, Beclin1 and LC3 II in the cells were measured. *p < 0.05, **p < 0.01, ***p < 0.001

Because FGF21 alleviates liver injury via autophagy and changes in SIRT1 expression, we focused on whether FGF21 attenuates ALI through autophagy mediated by SIRT1. We used SIRT1‐RNAi lentivirus to inhibit the expression of SIRT1 in mice and L02 cells. After confirmation of the desired effect of viral transfection (Figure S2), we first measured autophagy levels. Western blotting showed that levels of LC3 II and Beclin1 in the liver of the mice after SIRT1 knockdown were significantly lower by 1.54 and 1.32 times, respectively, compared with the FGF21‐treated group (Figure 5A–C). In line with this result, the immunohistochemical and immunofluorescence staining also showed that hepatic levels of LC3 II and Beclin 1 were significantly lower (Figure 5D–F). Similarly, in vitro assays showed that the levels of LC3 II and Beclin1 after SIRT1 knockdown were significantly lower by 1.42 and 1.36 times respectively (Figure 5G–I).

**FIGURE 6:**
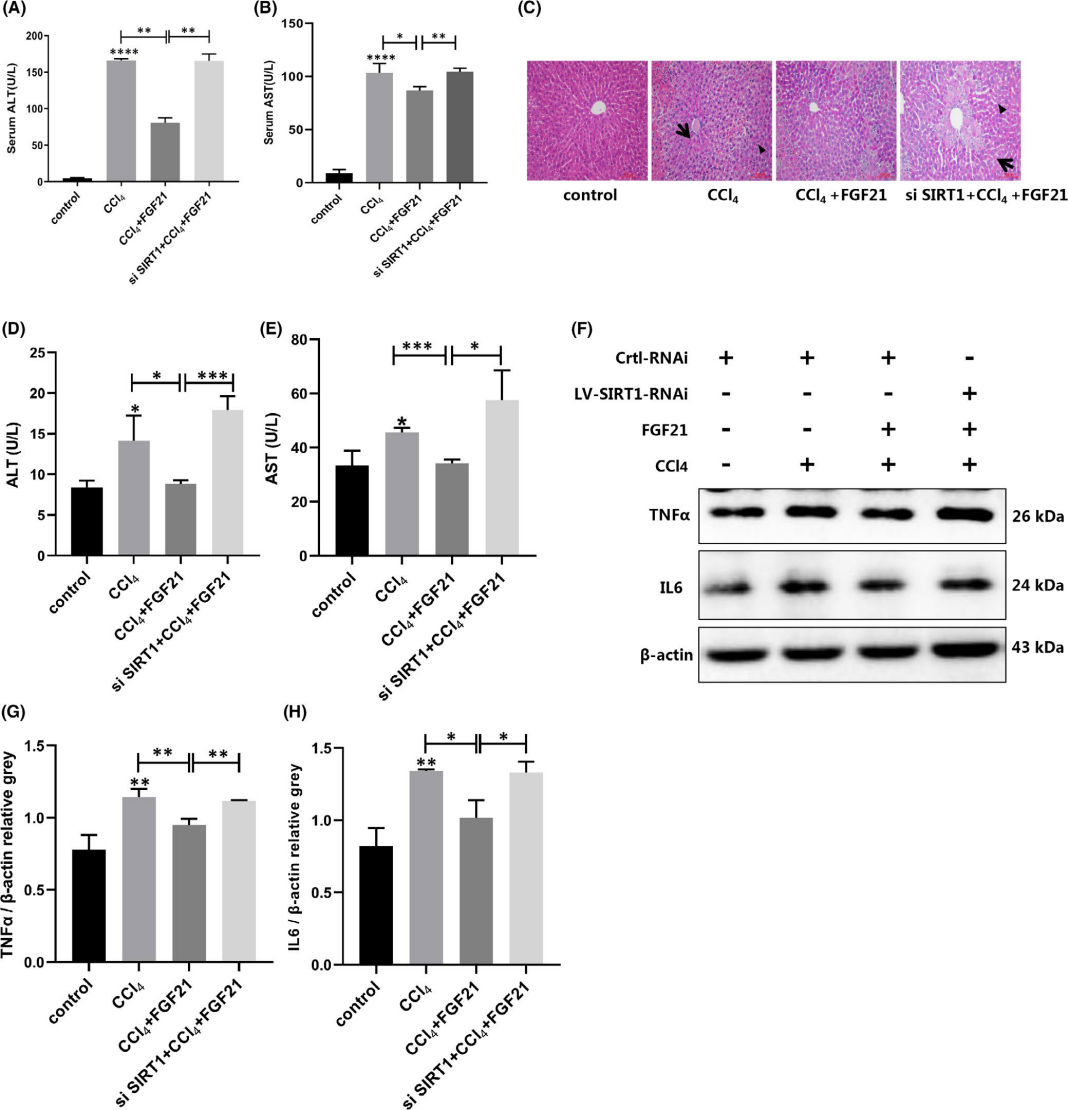
The downregulation of SIRT1 mediated by siRNA in liver or hepatocytes significantly reversed the ameliorative effect of FGF21 on ALI. (A and B) Mice were injected with siRNA‐mediated SIRT1 lentivirus, then CCl_4_ and FGF21 were given to the corresponding groups. We then measured serum transaminase levels. (C) Haematoxylin and eosin staining of the liver (scale bar = 100 µm). (D and E) After transfecting L02 cells with siRNA‐mediated SIRT1 lentivirus, the corresponding groups were treated with CCl_4_ and/or FGF21, and then we measured serum transaminase levels. (F and G) The expression of IL‐6 and TNF‐α in the cells. *p < 0.05, **p < 0.01, ***p < 0.001

Because the ability of FGF21 to induce autophagy was weakened after SIRT1 knockdown, we investigated whether its ability to alleviate liver injury was also diminished after SIRT1 knockdown. We found that the ability of FGF21 to ameliorate destroyed liver tissue and L02 cells in vitro after SIRT1 expression was inhibited. Transaminase levels increased 1.21–2.05‐fold as compared with the FGF21‐treated group. That is, the ability of FGF21 to reduce transaminases was significantly reversed (Figure 6A,B). Haematoxylin and eosin staining of the liver also showed that the liver structure was irregular, with more pronounced inflammatory infiltration, and aggravated liver damage after SIRT1 knockdown (Figure 6C). In vitro assays also showed that the effects of FGF21 treatment on reducing the levels of intracellular transaminases, IL‐6 and TNF‐α were no longer significant after SIRT1 knockdown; indeed, their expression levels were dramatically elevated again compared with those in the FGF21 group (Figure 6D–H). These results suggest that FGF21 alleviates ALI by inducing autophagy via regulating SIRT1 expression.

## 4 DISCUSSION

Liver disease is one of the most significant diseases threatening human health and is widespread in the population because of its prevalence and diversity. Nevertheless, treatment options for liver injury are limited. The effect of medications and their timing of action are usually limited. Moreover, liver transplantation is limited by the age of patients, complications such as infection, the difficultly treating graft‐versus‐host disease and high mortality. Due to these limitations, clinical treatment effects usually cannot meet patients' needs.^23^ Therefore, it is essential to find other new effective medications to alleviate liver injury.

FGF21 is 1 of 22 members of the fibroblast growth factor family. Unlike the classical members of FGF family, FGF21 does not possess heparin‐binding properties, which enables it to be released into the circulation.^24^ As an endocrine hormone, it profoundly affects systemic metabolism and participates in liver protection.^25^, ^26^ Moreover, evidence from both animal and clinical studies supported the role of FGF21 as a liver safeguard.^27^ Studies have reported that FGF21‐knockout mice consuming a lipotoxic diet show exacerbated liver damage, which can be prevented by exogenous FGF21 infusion.^28^, ^29^ In the present study, we first measured FGF21 expression during CCl_4_‐induced ALI. FGF21 expression significantly increased, with elevated expression in the most severely damaged parts of liver tissue; these findings suggest that FGF21 is activated rapidly and participates in ALI in mice. Experiments in vitro also showed that the FGF21 increases were most pronounced at 1 and 2 h after L02 cell injury, while the biological effect of FGF21 was no longer evident when the injury worsened, suggesting that FGF21 might protect against the early stages of ALI. To investigate the potential role of FGF21 in CCl_4_‐induced ALI, we next administered exogenous FGF21 to mice and L02 cells and found that the damage to liver tissue and the cells remarkably improved, suggesting that FGF21 alleviated CCl_4_‐induced ALI.

Next, we focused on the precise pathway by which FGF21 alleviates CCl_4_‐induced ALI. Autophagy is essential for maintaining cellular homeostasis by removing misfolded large molecules and dysfunctional organelles.^30^, ^31^ It often plays a protective role in the process of liver injury caused by various factors. Therefore, we considered the expression of autophagy in the process of FGF21 improvement of ALI. We found that increasing doses of FGF21 gave rise to an increased degree of autophagy. With our previous evidence on the FGF21 dose‐dependent effects on liver injury in the ALI group, these findings suggest that the alleviating effect of FGF21 is achieved by increasing the levels of autophagy in the liver. Furthermore, in vitro experiment confirmed this suggestion.

The process of FGF21 alleviation of ALI is related to autophagy; therefore, we determined how FGF21 affects autophagy and thereby alleviates ALI. SIRT1 is a vital histone deacetylase. SIRT1 and its activators play a pivotal role in cellular senescence, mitochondrial biogenesis, energy homeostasis and autophagy, in addition to effects against inflammation and oxidative stress.^32^ It has been reported that SIRT1 can regulate transcription factors, such as Nrf2 and NF‐κB, which are involved in the regulation of antioxidant genes in the face of oxidative damage and suppression of proinflammatory cytokines respectively.^33^, ^34^, ^35^, ^36^ Moreover, it has been reported that SIRT1‐deficient mice are highly susceptible to sepsis‐induced lung injury and overexpression of SIRT1 alleviated murine sepsis induced by LPS.^37^, ^38^ A study confirmed that deacetylation of LC3 II with SIRT1 is crucial for initiating autophagy.^39^ Previous studies have reported that autophagy mediated by SIRT1 plays a vital role in 2‐methoxyestradiol‐mediated prevention of ischemia/reperfusion injury in alcoholic fatty liver,^40^ and berberine protects against ischemia/reperfusion injury after orthotopic liver transplantation via activation of Sirt1/FoxO3a‐induced autophagy.^41^ Upregulation of SIRT1 attenuates ischemic liver injury and enhances mitochondrial recovery and autophagy.^42^ Therefore, we measured SIRT1 expression in the context of FGF21 treatment of ALI. The expression of SIRT1 was significantly upregulated while FGF21 induced autophagy, and the change in its expression showed an obvious similarity with autophagy. Next, we explored how SIRT1 expression affected autophagy expression, thereby affecting the therapeutic effect of FGF21 in alleviating ALI. We found that the ability of FGF21 to alleviate liver damage was significantly rescued after SIRT1 lentivirus was used to inhibit the expression of SIRT1 in mouse livers. Our in vitro experiment also showed that the inhibition of SIRT1 expression significantly inhibited the ability of FGF21 to induce autophagy. These findings suggest that FGF21 induces autophagy and alleviates ALI by promoting SIRT1 expression.

In conclusion, our findings suggest that FGF21 could alleviate CCl_4_‐induced acute liver injury through inducible expression of autophagy which is specifically mediated by SIRT1. The molecular mechanism of how FGF21 regulates the expression of SIRT1 and thereby affects autophagy needs to be further studied.

## CONFLICT OF INTEREST

The authors declare no conflict of interest.

## AUTHOR CONTRIBUTIONS

**Xiaoning Yang:** Conceptualization (equal). **Jin Zhongqian:** Data curation (equal). **Lin Danfeng:** Data curation (equal). **Shen Tianzhu:** Conceptualization (equal). **Zhang Jiangnan:** Conceptualization (equal). **Li Dan:** Formal analysis (equal). **Wang Xuye:** Formal analysis (equal). **Chi Zhang:** Writing – original draft (equal). **Zhuofeng Lin:** Writing – original draft (equal). **Xiaokun Li:** Writing – review & editing (equal). **Gong Fanghua:** Writing – review & editing (equal).

## Supporting information

Fig S1

Fig S2

## Data Availability

The data sets generated and/or analysed during the current study are available from the corresponding author on reasonable request.
