# Supplementary figures and images for: FGF21 alleviates acute liver injury by inducing the SIRT1‐autophagy signalling pathway
Source: J Cell Mol Med. 2022 Jan 4;26(3):868–79. doi: 10.1111/jcmm.17144 (PMC8817117; doi:10.1111/jcmm.17144)

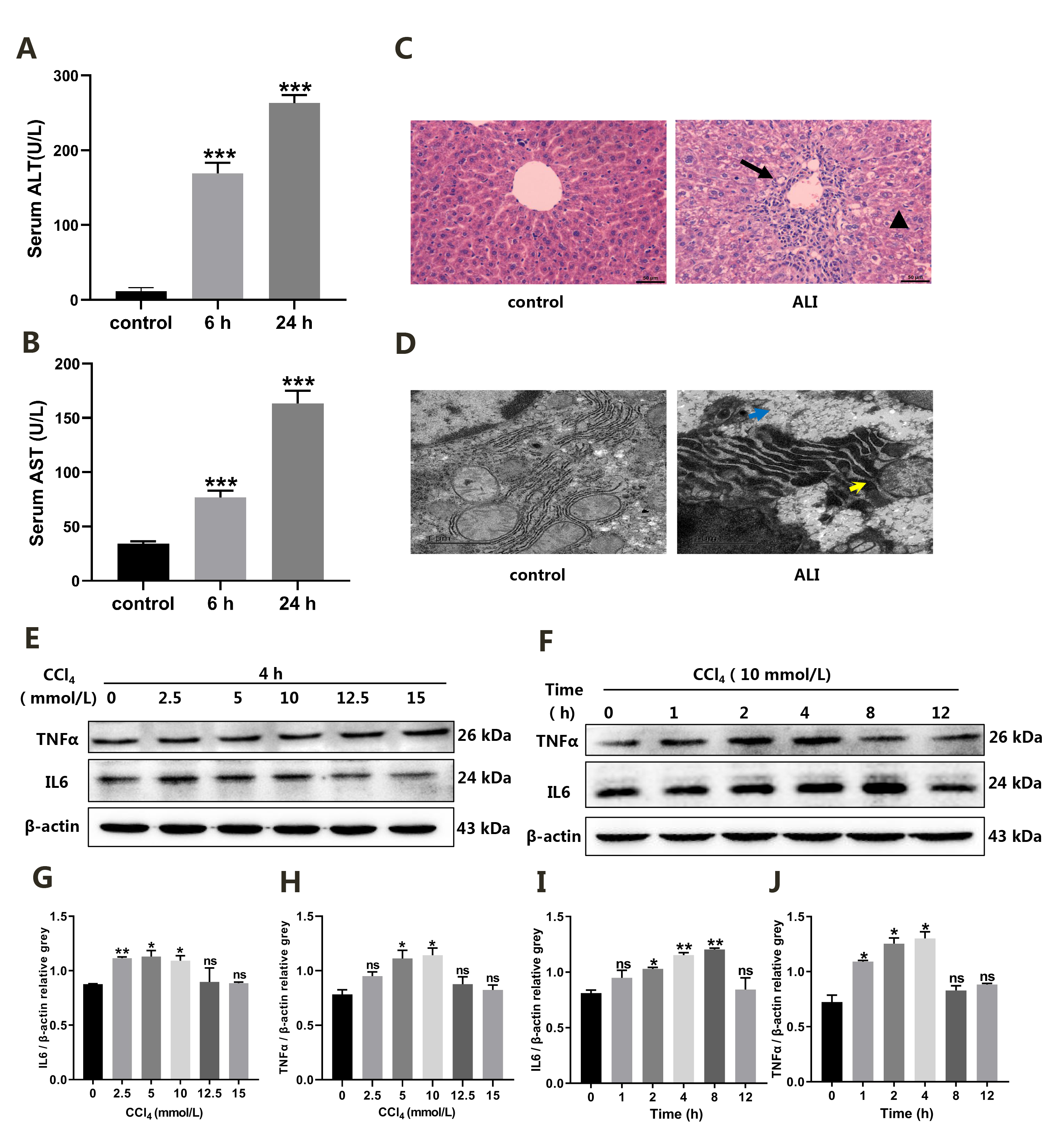

Supplement: Supplementary file 1 — Fig S1 [file JCMM-26-868-s001.tif]

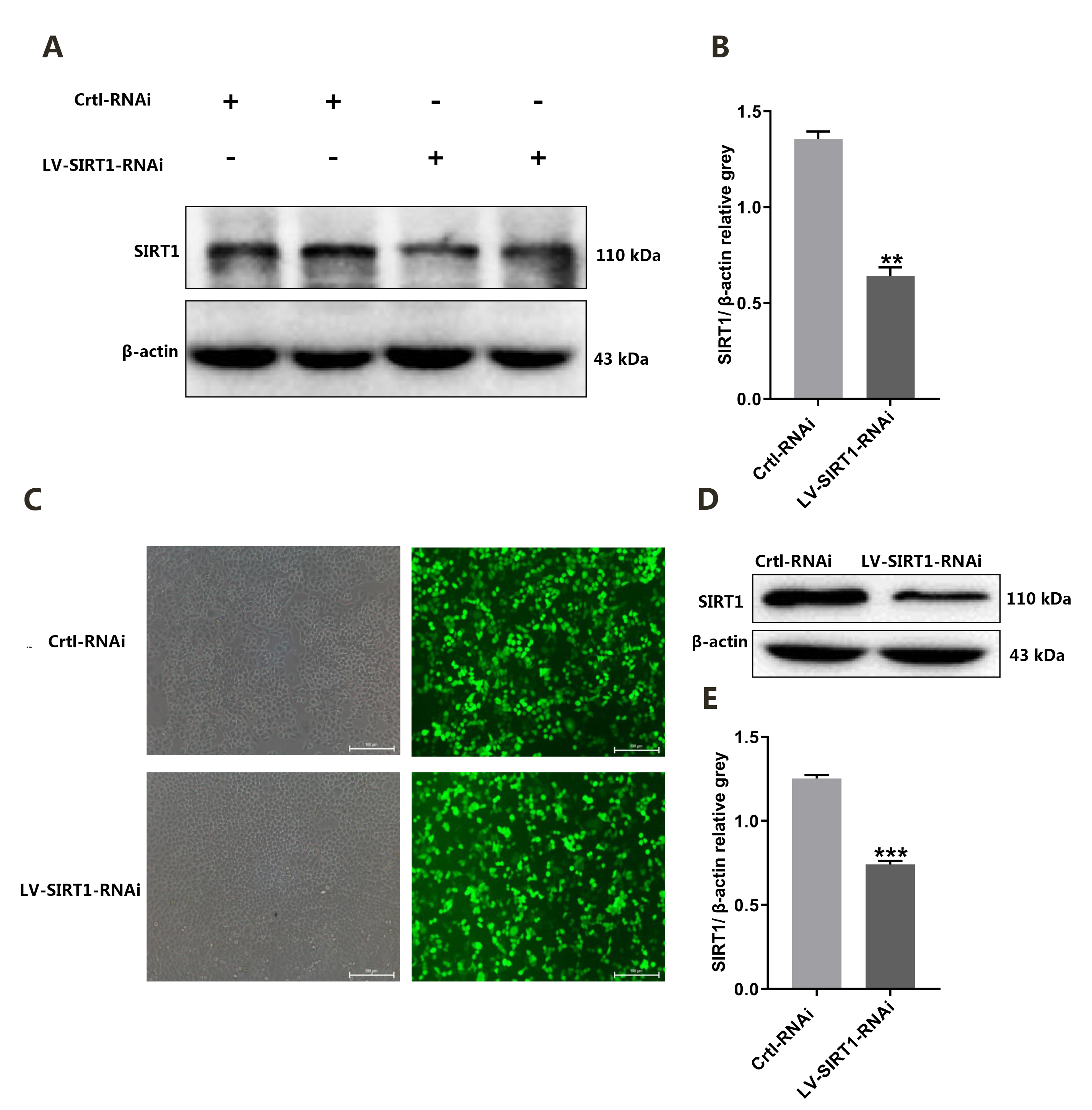

Supplement: Supplementary file 2 — Fig S2 [file JCMM-26-868-s002.tif]
